# Wild and cultivated allele effects on rice phenotypic traits in reciprocal backcross populations between *Oryza rufipogon* and two cultivars, *O. sativa* Nipponbare and IR36

**DOI:** 10.1270/jsbbs.22095

**Published:** 2023-09-09

**Authors:** Phuong Dang Thai Phan, Akinori Nishimura, Chika Yamamoto, Pham Thien Thanh, Toshihiro Niwa, Yaddehige Priya Jayantha Amarasinghe, Ryo Ishikawa, Takashige Ishii

**Affiliations:** 1 Graduate School of Agricultural Science, Kobe University, 1-1 Rokkodai, Nada-ku, Kobe, Hyogo 657-8501, Japan; 2 Research Institute for Biotechnology and Environment, Nong Lam University, Ho Chi Minh, Vietnam; 3 Food Crops Research Institute, Hai Duong, Vietnam

**Keywords:** backcross recombinant inbred lines, cultivated rice, quantitative trait locus (QTL) analysis, reciprocal genetic backgrounds, wild rice

## Abstract

A total of four populations of reciprocal backcross recombinant inbred lines were produced from a cross between a wild accession of *Oryza rufipogon* W630 and two major cultivars, *O. sativa* Japonica Nipponbare and Indica IR36. Using these populations, quantitative trait locus (QTL) analysis for eight morphological traits (culm length, panicle length, days to heading, panicle shape, pericarp color, hull color, seed shattering and seed awning) was carried out, and the putative QTL regions were compared among the populations. The QTLs with strong allele effects were commonly detected for culm length, panicle shape, pericarp color and hull color in all four populations, and their peak locations were close to the major genes of *sd1*, *Spr3*, *Rc* and *Bh4*, respectively. For panicle length and days to heading, some QTL regions overlapped between two or three populations. In the case of seed shattering and seed awning, strong wild allele effects at major loci were observed only in the populations with cultivated backgrounds. Since the wild and cultivated alleles have never been evaluated in the reciprocal genetic backgrounds, the present results provide new information on gene effects in breeding and domestication studies.

## Introduction

The genus *Oryza* consists of two cultivated species and more than 20 wild species (Vaughan 1994). Among them, the Asian wild species of *O. rufipogon* Griff. is an ancestral species of the common rice, *O. sativa* L. (Oka 1988). As this wild species readily crosses with the cultivated rice, many studies have identified useful genes and domestication-related genes using the segregating populations.

For example, dominant genes for grassy stunt virus resistance and blast resistance were transferred to rice cultivars from *O. nivara*, the annual form of *O. rufipogon*, (Ballini *et al.* 2007, Khush and Ling 1974), which are applied in rice breeding programs. Regarding domestication-related traits, seed-shattering behavior was found to be mainly controlled by a wild rice allele at *sh4* (Li *et al.* 2006), and seed awning was associated with three major loci of *An-1*, *LABA1* and *RAE2* (Bessho-Uehara *et al.* 2016, Hua *et al.* 2015, Luo *et al.* 2013). These traits are controlled by dominant genes in wild rice and can be phenotypically identified in the genetic background of cultivated rice. On the other hand, quantitative traits such as yield and plant size are associated with many genes, and their manner of inheritance is very complicated. Previously, wild alleles at some loci were estimated for high yield by QTL analysis (Xiao *et al.* 1996), however, wild responsible genes have not been identified so far. These results indicate that wild allele effects are different according to the traits and related loci.

In the genetic background of wild rice, allele replacement does not always affect wild phenotypes. In the case of seed-shattering behavior, cultivated loss-of-function alleles at *sh4* did not cause non-shattering phenotypes in wild rice (Ishikawa *et al.* 2010). Also, at seed awning loci *An-1*, *LABA1* and *RAE2*, cultivated alleles did not strongly influence awn length reduction in wild rice (Amarasinghe *et al.* 2020a, Ikemoto *et al.* 2017). Some other minor loci are probably associated with these traits in the genetic background of wild rice, suggesting that the allele effects may differ between the respective genetic backgrounds of wild rice and cultivated rice.

Gene introgression is the incorporation of a gene of one species into the gene pool of another. This process is usually generated by backcrossing with one parental species after interspecific hybridization. In rice breeding, gene introgression from wild species is important for transferring useful traits into rice cultivars (Jena 2010). In domestication studies, the performance of cultivated loss-of-function alleles in the wild genetic background is useful for understanding the domestication process (Konishi *et al.* 2006, Li *et al.* 2006). Therefore, it is necessary to clarify the association between phenotypic traits and loci in the different genetic backgrounds. In this study, we produced reciprocal backcross populations between *O. rufipogon* and two major rice cultivars, *O. sativa* Japonica Nipponbare and Indica IR36, and QTL analysis was carried out to examine the wild and cultivated allele effects on several qualitative and quantitative traits. Since the wild and cultivated allele effects have never been evaluated in the reciprocal genetic backgrounds, the present results will provide new information for both studies on breeding and domestication.

## Materials and Methods

### Plant materials

**Fig. 1.**
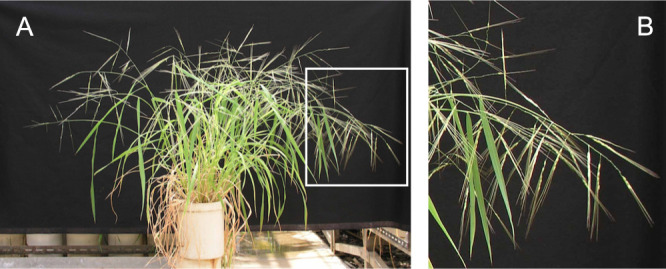
Plant morphology of *O. rufipogon* W630. (A) A whole plant at maturing stage. (B) An enlarged image of the white box in (A). Awned mature seeds are dispersed from the spreading panicles.

**Fig. 2.**
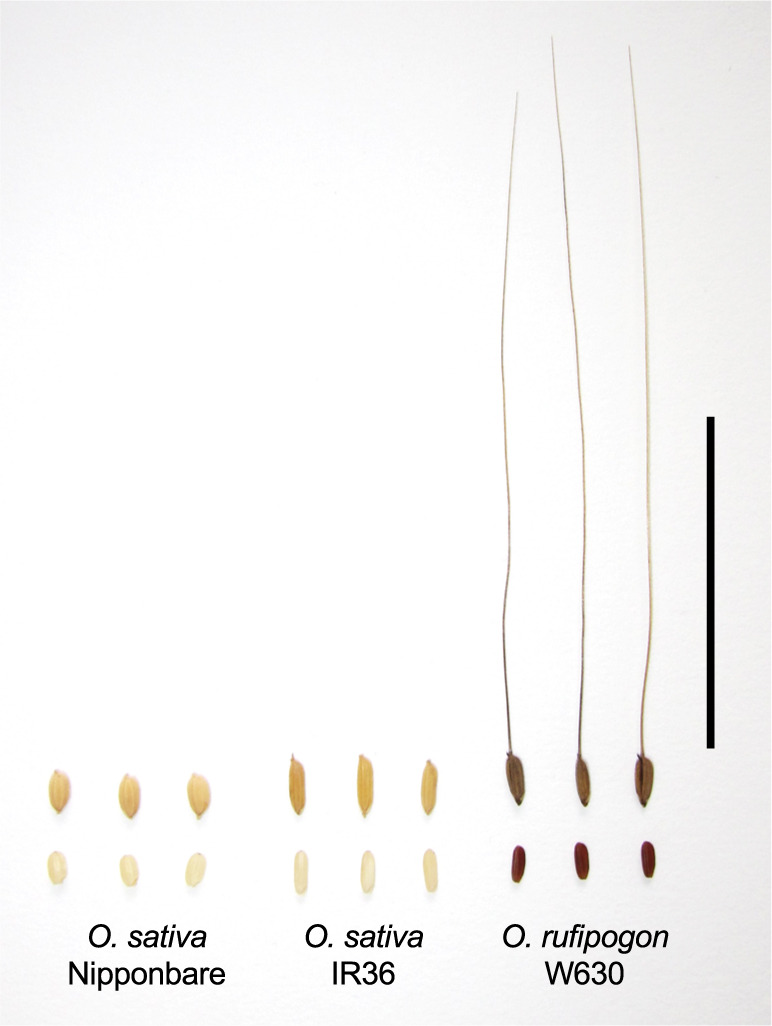
Seed morphology of *O. sativa* Nipponbare and IR36 and *O. rufipogon* W630. Three each of mature seeds (up) and dehulled seeds (down) were shown. Nipponbare and IR36 have white pericarp covered with straw-colored hulls, while wild pericarp and hull colors are red and black, respectively. Scale bar: 5 cm.

Two cultivars of *O. sativa* Japonica Nipponbare and Indica IR36, and a wild accession of *O. rufipogon* W630 were used in this study. Nipponbare and IR36 are popular Japonica and Indica rice cultivars, respectively. *O. rufipogon* W630 is an annual accession originated from Myanmar. It produces awned spikelets on the spreading panicles and disperses the mature seeds (Fig. 1) (Amarasinghe *et al.* 2020b). Its pericarp and hull colors are red and black, respectively, while the two cultivars have straw-colored seeds with white pericarp (Fig. 2). The wild accession was kindly provided by the National Institute of Genetics, Japan.

**Fig. 3.**
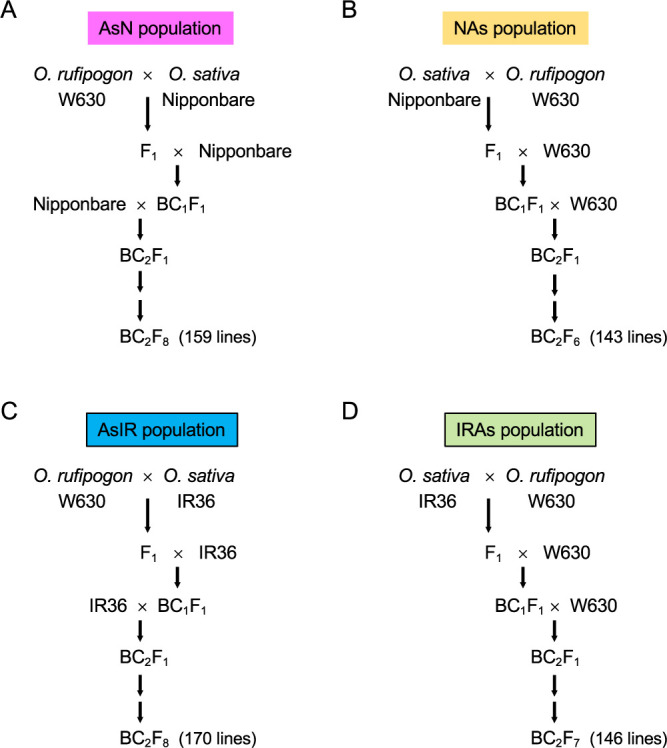
Four backcross recombinant inbred lines between *O. rufipogon* W630 and *O. sativa* Nipponbare and IR36. (A) AsN (donor parent: *O. rufipogon* W630, recurrent parent: *O. sativa* Nipponbare), (B) NAs (donor parent: *O. sativa* Nipponbare, recurrent parent:* O. rufipogon* W630), (C) AsIR (donor parent: *O. rufipogon* W630, recurrent parent: *O. sativa* IR36) and (D) IRAs (donor parent: *O. sativa* IR36, recurrent parent:* O. rufipogon* W630) populations. They consist of 159, 143, 170 and 146 lines, respectively.

The interspecific F_1_ plants were first produced between the wild accession and the two cultivars. They were reciprocally backcrossed twice to both parents, and the following four combinations of backcross recombinant inbred lines (BRILs) were developed by the single-seed-descendant method (Fig. 3). In this study, abbreviations of “N”, “IR” and “As” were given for Nipponbare, IR36 and Asian wild rice, *O. rufipogon* W630, respectively. In addition, “AsN” indicates Asian wild rice, *O. rufipogon* W630, backcrossed with *O. sativa* Nipponbare. Namely, the first and the last abbreviations show the donor and the recurrent parents, respectively.

AsN population: 159 lines at BC_2_F_8_ generation between W630 (donor parent) and Nipponbare (recurrent parent)

NAs population: 143 lines at BC_2_F_6_ generation between Nipponbare (donor parent) and W630 (recurrent parent)

AsIR population: 170 lines at BC_2_F_8_ generation between W630 (donor parent) and IR36 (recurrent parent)

IRAs population: 146 lines at BC_2_F_7_ generation between IR36 (donor parent) and W630 (recurrent parent)

### Trait evaluation

All four BRIL populations were grown in the paddy field of Kobe University, Japan. Their seedlings were transplanted in the first week of June. For AsN and AsIR populations with cultivated genetic backgrounds, they were planted in the plot with seven plants per row (20 cm between plants, 25 cm between rows). On the other hand, the seedlings of NAs and IRAs populations were planted at wider spacing of six plants per row (25 cm between plants, 30 cm between rows) because of their prostrate growth. After maturation, the following eight quantitative and qualitative traits were examined.

Culm length: Length from ground to the panicle neck of the main stem (cm).

Panicle length: Panicle length of the main stem (cm).

Days to heading: Days from seed soaking to heading of the first panicle.

Panicle shape: Scale of 0–2 (0: closed, 1: intermediate, 2: open).

Pericarp color: Scale of 0–2 (0: white, 1: intermediate, 2: red).

Hull color: Scale of 0–2 (0: straw, 1: intermediate, 2: black).

Seed shattering: Scale of 0–2 (0: non-shattering, 1: intermediate, 2: shattering) for AsN, NAs and IRAs populations. Average breaking tensile strength (gram-force: gf) value to detach a grain from the pedicel using a digital force gauge (FGP 0.5, Nidec-Shimpo Co., Japan) for AsIR population.

Seed awning: Scale of 0–3 (0: <1 mm, 1: 1–5 mm, 2: 5–15 mm, 3: 15 mm<) examined for average awn length of 1st spikelet on the top primary branch (n = 5).

### QTL analysis

In each population, young leaves were collected from the BRIL plants, from which their genomic DNA samples were isolated by a potassium acetate method (Dellaporta *et al.* 1983). In total, 177 polymorphic simple sequence repeat (SSR) markers between *O. sativa* Nipponbare and *O. rufipogon* W630 were used to examine BRIL genotypes in AsN and NAs populations (Supplemental Tables 1, 2). On the other hand, 163 and 156 polymorphic SSR markers between *O. sativa* IR36 and *O. rufipogon* W630 were used for AsIR and IRAs populations, respectively (Supplemental Tables 3, 4). Of these markers, 141 markers were common among the four populations. Primer sequences of the SSR markers were obtained from the Gramene database (Liang *et al.* 2008). After PCR, the amplified products were electrophoresed in 4% polyacrylamide denaturing gel. The microsatellite banding patterns were visualized using the silver staining method (Panaud *et al.* 1996).

Based on the marker and trait data, QTL analysis was carried out with molecular linkage map information after Thanh *et al.* (2010). For the metric trait data, a putative QTL was estimated by composite interval mapping with WinQTL Cartographer software version 2.5 (Wang *et al.* 2007). The optimal log of odds (LOD) threshold values were calculated by 1000 permutation tests. For the ordinal data (scored by scales), a single-marker regression analysis was carried out with QGene version 4.3 (Joehanes and Nelson 2008), and a putative QTL was estimated based on the threshold LOD value of 3.0.

## Results

### Percentages of introgressed chromosomal segments in the BRIL populations

The four BRIL populations were generated from the BC_2_F_1_ plants by the single-seed-descendant method (Fig. 3). Theoretically, the BRILs have 1/8 (12.5%) of the donor genome in the genetic background of the recurrent parent. Based on the marker genotypes at microsatellite loci across the 12 rice chromosomes (177, 177, 163 and 156 loci for AsN, NAs, AsIR and IRAs populations, respectively), the donor chromosomal segments in each line were estimated (Supplemental Tables 1–4). In the genetic background of the cultivated rice, the percentages of wild segments among lines ranged from 0.0% to 23.3% (average = 11.0%) and from 0.0% to 22.6% (average = 10.0%) in the AsN and AsIR populations, respectively (Supplemental Table 5). Some lines did not have donor alleles in the marker survey, but they showed different phenotypes from the recurrent parent. Therefore, they were included in the QTL analysis. On the other hand, the percentages of cultivated segments ranged from 1.1% to 31.3% (average = 11.5%) and from 1.3% to 30.5% (average = 14.9%) in the NAs and IRAs populations, respectively (Supplemental Table 5). These values are close to the theoretical percentage of 12.5%. In addition, donor alleles were detected at all SSR marker loci without gaps (Supplemental Tables 1–5). Several marker loci were found to have much less donor alleles (<2.5%), but their common chromosomal regions were not detected among four populations. Instead, RM273 on chromosome 4 showed the highest ratios of cultivated alleles in the wild genetic backgrounds; 24.5% in NAs and 42.8% in IRAs populations. At the same locus, the wild allele ratios were relatively low in AsN (5.0%) and AsIR (5.9%), suggesting that an advantageous chromosomal region for cultivated alleles might exist near the RM273 locus. The average homozygosity values were higher than 98.5% (Supplemental Table 5), indicating that the BRILs are almost fixed lines in the four populations.

### Phenotypic variation in the BRIL populations

**Fig. 4.**
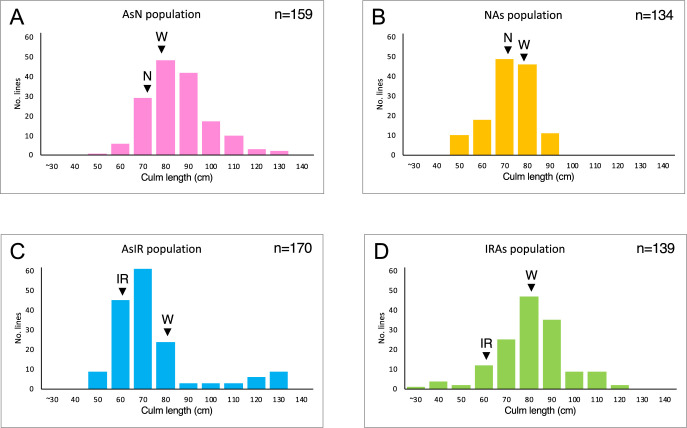
Frequency distributions of culm length in four backcross recombinant inbred lines between *O. sativa* and *O. rufipogon*. (A) AsN, (B) NAs, (C) AsIR and (D) IRAs populations. Parental phenotypic means are indicated by arrows. N: *O. sativa *Nipponbare, IR: *O. sativa* IR 36, W: *O. rufipogon* W630.

Fig. 4 shows the frequency distributions of culm length in the four BRIL populations. The distributions were discontinuous and almost bell-shaped. Several plants with a culm length greater than 120 cm were observed in the AsIR population. In all four populations, many lines had the values outside the range of the parent culm lengths. Discontinuous distributions were observed for panicle length and days to heading (Supplemental Figs. 1, 2). Transgressive variation was also detected in all the populations.

Panicle shape, pericarp color and hull color clearly differed between wild and cultivated rice plants. These domestication-related traits were measured in the scale of 0–2 (0: cultivar type, 1: intermediate type, 2: wild type) (Table 1). In the AsN and AsIR populations with cultivated genetic background, most of the lines showed cultivar phenotypes. Conversely, wild phenotypes were frequently observed in the NAs and IRAs populations with wild background.

Previously, we analyzed seed shattering and seed awning characters using the AsN population (Ikemoto *et al.* 2017, Ishikawa *et al.* 2010). These traits were further examined in other three populations (Table 1). In the NAs and IRAs populations with wild genetic background, almost all the lines showed wild phenotypes for seed shattering and seed awning. In the AsIR population, seed shattering was measured based on the average breaking tensile strength using 60 spikelets (20 spikelets from three panicles of each line) about seven weeks after flowering, and their average values were calculated (Supplemental Fig. 3). Seed awning was evaluated in the scale of 0–3 based on the awn length. A total of 63 lines were awnless (<1 mm), whereas the other 107 lines had seeds with different awn lengths (Table 1).

### QTL analysis for eight morphological traits in the BRIL populations

Based on the marker and trait data, QTL analysis was carried out with the linkage map information after Thanh *et al.* (2010). For the metric trait data, composite interval mapping was used to detect putative QTLs (Table 2). In the analysis of culm length, three, two, one and three QTLs were estimated for the AsN, NAs, AsIR and IRAs populations, respectively. Among them, one QTL on chromosome 1 was commonly detected with high LOD values. Regarding panicle length, one QTL was detected in each of the AsN and AsIR populations having the cultivated genetic background. On the other hand, five and four QTLs were estimated in the wild NAs and IRAs populations, respectively. Only a QTL detected on chromosome 7 in the NAs population had a LOD higher than 10. For days to heading, one to five QTLs were estimated in the four populations, however the positions of QTLs with the highest LOD values were different in each population. These QTLs were detected on chromosomes 3, 1, 8 and 6 in the AsN, NAs, AsIR and IRAs populations, respectively. For seed shattering in the AsIR population, two QTLs were estimated on chromosomes 3 and 4.

For the traits with ordinal data, a single-marker regression analysis was carried out to detect the level of linkage between the traits and markers (Table 3). In the analysis of panicle shape, pericarp color and hull color, at least one QTL was detected in each population. Of these, QTLs with the highest LOD values in the four populations were common for panicle shape (chromosome 4), pericarp color (chromosome 7) and hull color (chromosome 4). Regarding seed shattering and seed awning, two QTLs each were detected in the AsN and AsIR populations. No QTLs were estimated in the NAs and IRAs populations, because almost all the lines showed wild phenotypes.

In the AsN population, QTL analysis for days to heading, panicle shape, seed shattering and seed awning was re-examined using the data sets of Thanh *et al.* (2010), Ishii *et al.* (2013), Ishikawa *et al.* (2010) and Ikemoto *et al.* (2017), respectively.

## Discussion

In this study, putative QTLs were estimated for eight morphological traits using four BRIL populations between *O. rufipogon* and two typical rice cultivars, *O. sativa* Nipponbare and IR36. Since these four populations were derived from a single wild accession of W630, the candidate QTL regions are worth comparing to evaluate wild and cultivated allele effects in the different genetic backgrounds. The QTLs may be divided into two groups, as these candidate regions are assumed to be only in one population or are commonly overlapped in different populations.

**Fig. 5.**
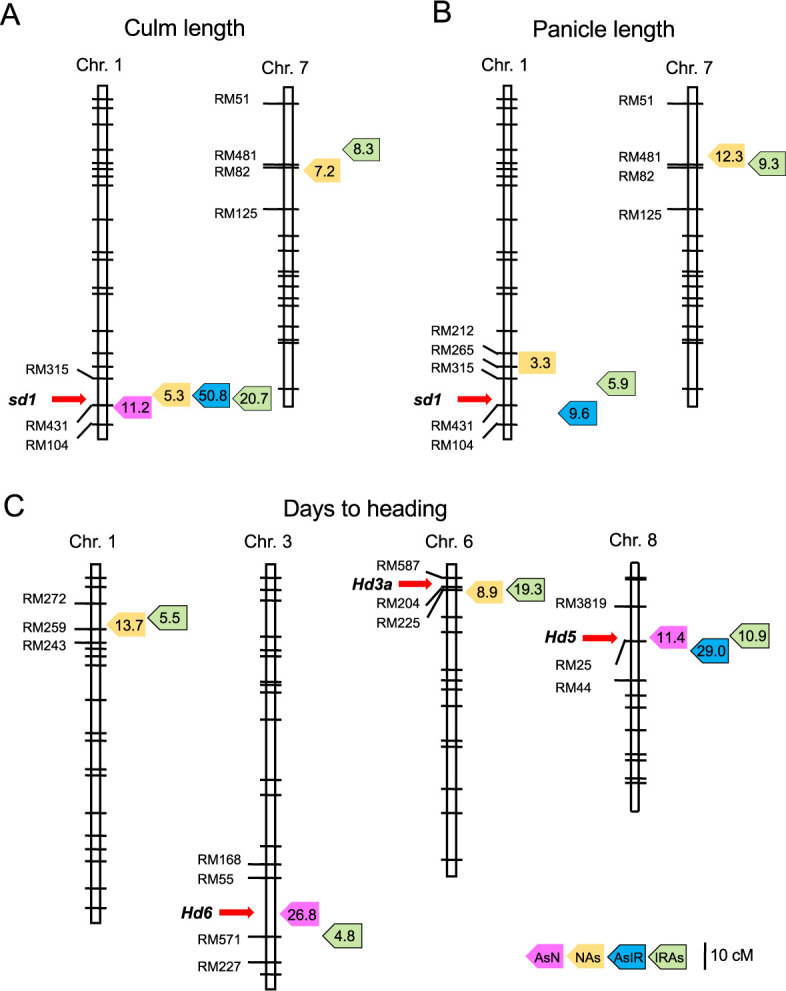
Putative QTL locations for three morphological traits commonly detected in the populations. (A) Culm length. (B) Panicle length. (C) Days to heading. Peak QTL positions are shown by colored boxes with LOD values: pink, orange, blue and green colors for AsN, NAs, AsIR and IRAs populations, respectively. Red arrows show the major genes for the traits.

For culm length, a total of nine QTLs were estimated in the four populations (Table 2). Of these, one on chromosome 1 was commonly detected with high LOD values in all populations (Fig. 5). In the candidate region, *sd1* locus is located near the peaks. At this locus, rice cultivars were reported to have obtained alleles controlling plant architecture in domestication (Asano *et al.* 2011). In addition, IR36 has a loss-of-function allele causing semi-dwarfism (Sasaki *et al.* 2002). Therefore, the cultivated and wild allele effects were strongly reflected in both genetic backgrounds. On chromosome 7, the candidate regions were overlapped in the NAs and IRAs populations. The cultivated alleles probably contribute short culm structure in the wild genetic background. Similarly, two QTL regions for panicle length on chromosomes 1 and 7 were shared between the populations (Fig. 5). This suggests that the *sd1* alleles may have influence on panicle length. The QTLs on chromosome 7 showed higher LOD values than those on chromosome 1, and the region was close to that detected for culm length. The cultivated alleles related to plant growth in the wild background may exist at this locus.

Many genes and loci have been reported for heading date in rice (Itoh *et al.* 2018, Nonoue *et al.* 2019). In this study, a total of 13 QTL regions were estimated on seven chromosomes in four populations (Table 2). Among them, four were overlapped in more than two populations (Fig. 5). Near the peaks of their LOD score, three known genes for heading date were identified: *Hd6*, *Hd3a* and *Hd5* on chromosomes 3, 6 and 8, respectively (Kojima *et al.* 2002, Takahashi *et al.* 2001, Wei *et al.* 2010). Notably, QTLs with the highest LOD values in each population were estimated in the different chromosomal regions (Table 2, Fig. 5). These suggest that heading date is associated with many loci, and that their allele effects are different depending on the genetic backgrounds.

**Fig. 6.**
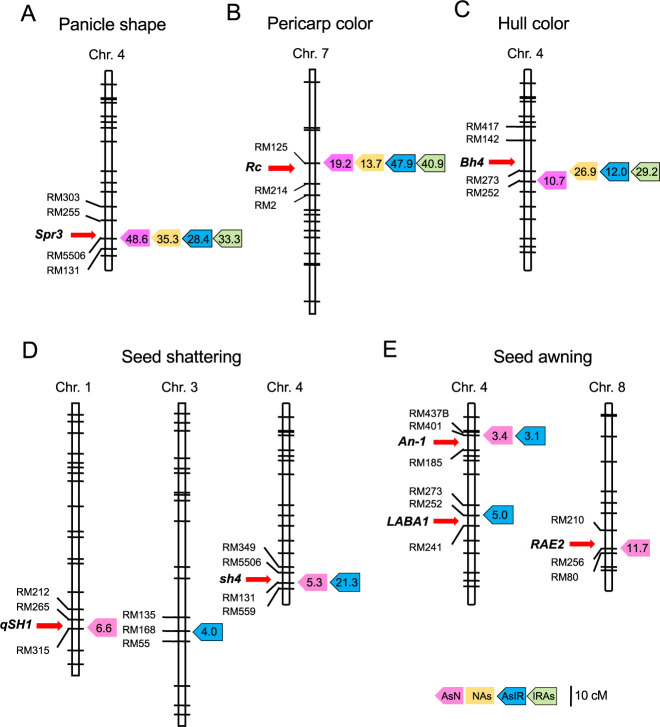
Putative QTL locations for five morphological traits commonly detected in the populations. (A) Panicle shape. (B) Pericarp color. (C) Hull color. (D) Seed shattering. (E) Seed awning. Peak QTL positions are shown by colored boxes with LOD values: pink, orange, blue and green colors for AsN, NAs, AsIR and IRAs populations, respectively. Red arrows show the major genes for the traits.

Several QTLs were associated with panicle shape, pericarp color and hull color, and the QTLs that explained the highest percentages of phenotypic variance were detected with common or closely linked markers (Table 3). Near the markers, *Spr3*, *Rc* and *Bh4* loci were found on chromosomes 4, 7 and 4, respectively (Fig. 6). In the previous studies, wild rice was reported to have functional alleles at these loci, while cultivated rice had null or weak alleles (Ishii *et al.* 2013, Sweeney *et al.* 2006, Zhu *et al.* 2011). These results indicate that the phenotypes of these three traits were mostly determined by the presence or absence of the functional alleles regardless of genetic background.

In the case of seed shattering and seed awning, loss-of-function mutations at some major loci are associated with cultivated phenotypes, i.e., non-seed shattering and awnless seed. This explains why typical cultivated phenotypes were hardly observed in the NAs and IRAs populations with wild genetic background. On the other hand, a few QTLs were detected in the genetic background of cultivated rice (Table 3). In rice, two major genes (*qSH1* and *sh4*) and three major genes (*An-1*, *LABA1* and *RAE2*) were identified for seed shattering and seed awning, respectively (Bessho-Uehara *et al.* 2016, Hua *et al.* 2015, Konishi *et al.* 2006, Li *et al.* 2006, Luo *et al.* 2013). Of these,* sh4* and *An-1 *loci were detected in both AsN and AsIR populations (Fig. 6). The *qSH1* and *RAE2* loci were found only in the AsN population, whereas the *LABA1* was estimated in the AsIR population. This may be due to the fact that the Nipponbare and IR36 alleles are functional at *LABA1* and at *qSH1* and *RAE2*, respectively (Amarasinghe *et al.* 2020a, Hua *et al.* 2015, Ishikawa *et al.* 2017).

In this study, QTLs for eight morphological traits were estimated using four BRIL populations derived from the reciprocal crosses between *O. rufipogon* and two typical rice cultivars, *O. sativa* Japonica Nipponbare and Indica IR36. By comparing the putative QTL regions, the wild and cultivated allele effects were investigated in both genetic backgrounds. In rice breeding, gene introgression from wild is important to transfer useful traits to cultivated Japonica as well as Indica gene pools. In domestication studies, the performance of Japonica and Indica loss-of-function alleles in the same wild genetic background is quite helpful to clarify their domestication process. Such comprehensive information can not be obtained from a single-cross population. Therefore, the present results on gene effects will be informative for both breeding and domestication studies.

## Author Contribution Statement

TI conceived and designed the research. PDTP, AN, CY, PTT and TN performed the field experiments. PDTP, AN, CY, PTT and YPJA analyzed the genotypic data. TI and RI provided the direction for the study and prepared the manuscript. All authors read and approved the final manuscript.

## Supplementary Material

Supplemental Figures

Supplemental Table 1

Supplemental Table 2

Supplemental Table 3

Supplemental Table 4

Supplemental Table 5

## Figures and Tables

**Table 1.** Segregation of five morphological traits (panicle shape, pericarp color, hull color, seed shattering and seed awing) with ordinal scales in four backcross recombinant inbred lines, AsN, NAs, AsIR and IRAs populations

Trait	Population	No. of lines					Total
		0	1	2	3	n.d.*^a^*	
Panicle shape	AsN	116	6	37		0	159
	NAs	13	0	126		4	143
	AsIR	134	14	22		0	170
	IRAs	32	0	114		0	146
Pericarp color	AsN	145	2	12		0	159
	NAs	10	13	119		1	143
	AsIR	141	12	17		0	170
	IRAs	20	5	121		0	146
Hull color	AsN	155	4	0		0	159
	NAs	5	43	93		2	143
	AsIR	154	6	10		0	170
	IRAs	3	65	77		1	146
Seed shattering	NAs	0	1	141		1	143
	IRAs	0	2	144		0	146
Seed awning	NAs	0	0	0	143	0	143
	AsIR	63	48	41	18	0	170
	IRAs	0	0	0	146	0	146

*^a^* Not determined.

**Table 2.** Putative QTLs for the four traits (culm length, panicle length, days to heading and seed shattering) with metric data detected in four backcross recombinant inbred lines, AsN, NAs, AsIR and IRAs populations

Trait	Population	Chr.	Location	Nearest marker	LOD	PV*^a^*	Additive effect*^b^*	Reference
Culm length (cm)	AsN	1	RM315 - RM104	RM431	11.2	19.3	9.6	
		4	RM255 - RM559	RM5506	9.6	17.2	7.2	
		5	RM440 - RM538	RM173	9.4	17.6	8.3	
	NAs	1	RM315 - RM104	RM431	5.3	18.2	6.9	
		7	RM51 - RM125	RM82	7.2	17.6	–6.2	
	AsIR	1	RM315 - RM431	RM431	50.8	75.2	26.3	
	IRAs	1	RM84 - RM272	RM1	5.0	8.8	–6.4	
		1	RM315 - RM104	RM431	20.7	42.3	20.4	
		7	RM51 - RM125	RM481	8.3	19.9	–10.2	
Panicle length (cm)	AsN	5	RM440 - RM538	RM421	5.3	13.8	1.7	
	NAs	1	RM212 - RM315	RM265	3.3	6.9	1.1	
		3	RM7 - RM282	RM251	3.3	8.2	1.3	
		4	RM252 - RM241	RM241	3.2	5.6	–0.8	
		6	RM162 - RM412	RM528	5.3	16.5	1.6	
		7	RM451 - RM125	RM427	12.3	36.2	–2.7	
	AsIR	1	RM315 - RM104	RM431	9.6	21.9	3.8	
	IRAs	1	RM272 - RM23	RM243	7.1	15.2	–1.6	
		1	RM315 - RM104	RM315	5.9	14.0	2.9	
		7	RM51 - RM125	RM481	9.3	15.5	–2.0	
		10	RM474 - RM244	RM222	5.2	11.3	1.6	
Days to heading	AsN	2	RM221 - RM240	RM6	4.1	5.1	2.8	Thanh *et al.* (2010)
		3	RM135 - RM227	RM571	26.8	44.1	9.0	Thanh *et al.* (2010)
		8	RM3819 - RM44	RM25	11.4	18.3	5.4	Thanh *et al.* (2010)
		10	RM484 - RM228	RM228	7.0	9.0	4.0	Thanh *et al.* (2010)
	NAs	1	RM272 - RM580	RM259	13.7	36.9	–5.8	
		6	RM587 - RM253	RM225	8.9	17.6	–8.2	
		12	RM463 - RM235	RM235	3.7	6.7	2.1	
	AsIR	8	RM3819 - RM44	RM25	29.0	58.7	13.7	
	IRAs	1	RM272 - RM243	RM259	5.5	7.7	–2.3	
		3	RM55 - RM227	RM571	4.8	5.2	–2.5	
		6	RM133 - RM253	RM204	19.3	26.0	–12.3	
		6	RM253 - RM3	RM539	17.3	23.7	5.1	
		8	RM38 - RM44	RM25	10.9	18.1	5.0	
Seed shattering (gf)	AsIR	3	RM411 - RM55	RM168	4.0	7.2	–8.2	
		4	RM255 - RM559	RM131	21.3	44.2	–16.9	

*^a^* Percentage of the phenotypic variance explained by the QTL. *^b^* Additive effect of the wild allele.

**Table 3.** Putative QTLs for the five traits (panicle shape, pericarp color, hull color, seed shattering and seed awning) with ordinal data detected in four backcross recombinant inbred lines, AsN, NAs, AsIR and IRAs populations

Trait	Population	Chr.	Location	Nearest marker	LOD	PV*^a^*	Reference
Panicle shape	AsN	4	RM241 - RM559	RM5506	48.6	75.5	Ishii *et al.* (2013)
	NAs	2	RM53 - RM8	RM8	5.8	17.4	
		4	RM255 - RM559	RM5506	35.3	68.9	
	AsIR	4	RM273	RM273	3.7	9.5	
		4	RM303 - RM559	RM5506	28.4	53.7	
	IRAs	4	RM273 - RM559	RM5506	33.3	65.0	
		9	RM205	RM205	3.1	9.4	
Pericarp color	AsN	1	RM259	RM259	3.3	9.1	
		4	RM273 - RM241	RM273	4.8	12.9	
		7	RM125 - RM346	RM125	19.2	42.7	
	NAs	1	RM9 - RM246	RM5	6.3	18.6	
		4	RM417	RM417	4.1	12.5	
		7	RM125 - RM2	RM125	13.7	35.8	
	AsIR	7	RM481 - RM2	RM125	47.9	72.6	
	IRAs	7	RM51 - RM346	RM125	40.9	72.5	
Hull color	AsN	4	RM142 - RM241	RM252	10.7	26.7	
		7	RM82 - RM125	RM125	8.4	21.6	
	NAs	4	RM185 - RM303	RM273	26.9	58.4	
	AsIR	4	RM273 - RM303	RM273	12.0	27.7	
		7	RM214	RM214	3.0	7.8	
	IRAs	4	RM273 - RM131	RM273	29.2	60.4	
		10	RM258	RM258	3.2	9.5	
Seed shattering	AsN	1	RM212 - RM315	RM315	6.6	15.2	Ishikawa *et al.* (2010)
		4	RM349 - RM131	RM131	5.3	12.3	Ishikawa *et al.* (2010)
Seed awning	AsN	4	RM437B - RM401	RM401	3.4	9.5	Ikemoto *et al.* (2017)
		8	RM223 - RM502	RM256	11.7	28.8	Ikemoto *et al.* (2017)
	AsIR	4	RM401	RM401	3.1	8.0	
		4	RM273 - RM241	RM252	5.0	12.6	

*^a^* Percentage of the phenotypic variance explained by the QTL.
