# Supplementary material for: Wild and cultivated allele effects on rice phenotypic traits in reciprocal backcross populations between Oryza rufipogon and two cultivars, O. sativa Nipponbare and IR36
Source: Breed Sci. 2023 Sep 9;73(4):373–81. doi: 10.1270/jsbbs.22095 (PMC10722096; doi:10.1270/jsbbs.22095)
Supplement: Supplementary file 1 — Supplemental Figures [file 73_373_s1.pdf]

Phan et al./Breeding Science
doi: 10.1270/jsbbs.22095

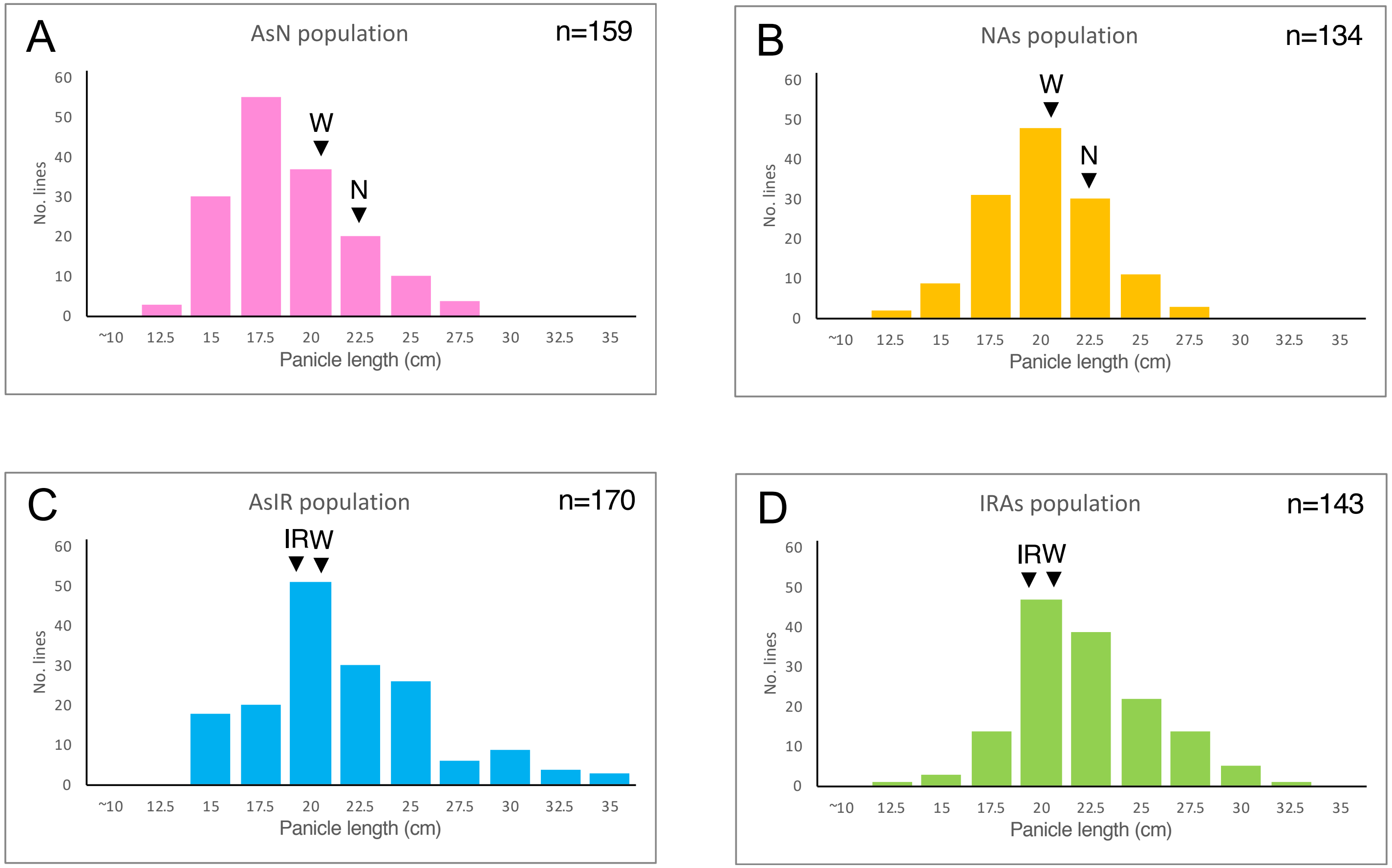

Supplemental Fig. 1. Frequency distributions of panicle length in four backcross recombinant inbred lines between *O. sativa* and *O. rufipogon*. (A) AsN, (B) NAs, (C) AsIR and (D) IRAs populations. Parental phenotypic means are indicated by arrows. N: *O. sativa* Nipponbare, IR: *O. sativa* IR36, W: *O. rufipogon* W630.

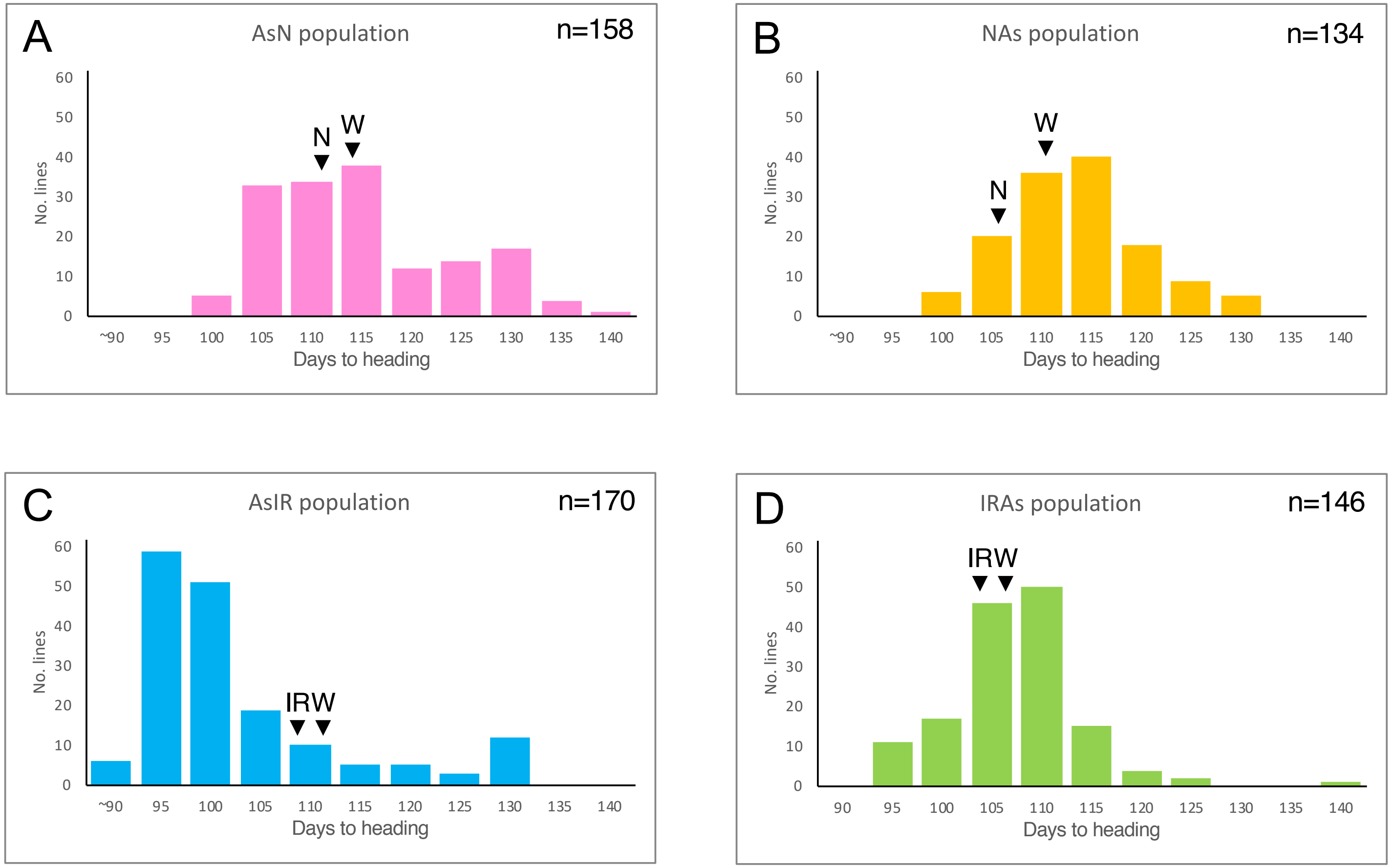

Supplemental Fig. 2. Frequency distributions of days to heading in four backcross recombinant inbred lines between *O. sativa* and *O. rufipogon*. (A) AsN, (B) NAs, (C) AsIR and (D) IRAs populations. Parental phenotypic means are indicated by arrows. N: *O. sativa* Nipponbare, IR: *O. sativa* IR36, W: *O. rufipogon* W630.

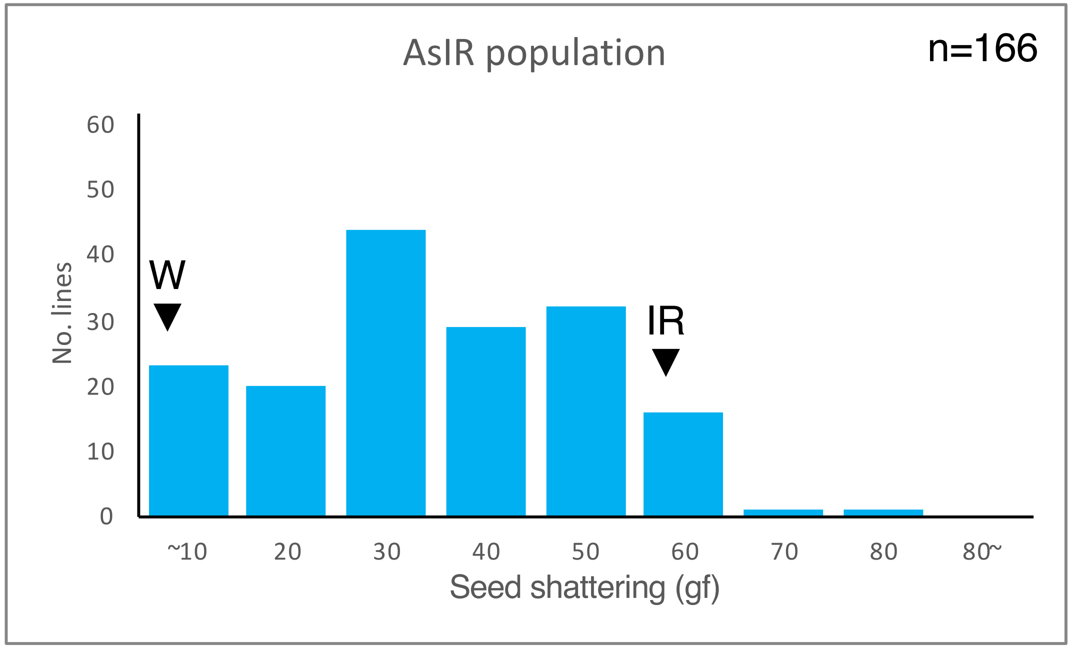

Supplemental Fig. 3. Frequency distributions of seed shattering degree in AsIR population between *O. sativa* IR36 and *O. rufipogon*. Parental phenotypic means are indicated by arrows. IR: *O. sativa* IR36, W: *O. rufipogon* W630.
